# Patient Follow-up After Endovascular Treatment of Cerebral Aneurysms: Identifiable Barriers and Implications for Treatment Decisions

**DOI:** 10.1227/neuprac.0000000000000174

**Published:** 2025-10-13

**Authors:** Zachary A. Sorrentino, Hunter Hutchinson, Chloe L. DeYoung, Ivy Li, Brandon Lucke-Wold, Danyas Sarathy, Arnold Obungu, Nohra Chalouhi, Brian L. Hoh, Matthew J. Koch

**Affiliations:** ‡ University of Florida College of Medicine, Gainesville, Florida, USA;; § Department of Neurosurgery, University of Florida College of Medicine, Gainesville, Florida, USA

**Keywords:** Subarachnoid hemorrhage, Cerebral aneurysm, Endovascular treatment, Clipping, Socioeconomic status

## Abstract

**BACKGROUND AND OBJECTIVES:**

Cerebral aneurysms are the major cause of spontaneous subarachnoid hemorrhage, and a common treatment is endovascular embolization. Aneurysmal recurrence after embolization is frequent, and clinical follow-up to monitor for this is critical to prevent subarachnoid hemorrhage. Herein, we assess demographic and socioeconomic factors associated with poor clinical follow-up to determine which patients are at high risk of loss to follow-up.

**METHODS:**

A retrospective analysis was performed of 937 patients who underwent endovascular treatment of cerebral aneurysms at a single center from 2006 to 2017. Attendance at follow-up visits for 5 years after treatment was correlated with various demographic and socioeconomic factors. Follow-up for patients who initially presented with unruptured vs ruptured aneurysms was also compared.

**RESULTS:**

The most significant factors associated with poor follow-up were lack of insurance and non–English-speaking status. Noninsured patients had significantly less follow-up in both mean number of follow-up visits (2.5 ± 1.7 visits for insured patients and 1.0 ± 1.2 visits for noninsured, *P* < .0001), and in time until lost to follow-up (2.9 ± 1.9 years for insured and 1.1 ± 1.4 years for non-insured, *P* < .0001). Non–English-speaking patients had less follow-up visits (2.3 ± 1.7 visits for English-speaking and 0.74 ± 1.0 visits for non–English-speaking, *P* < .0001) and were more quickly lost to follow-up (2.6 ± 1.9 years for English-speaking and 0.82 ± 1.2 years for non–English-speaking, *P* < .0001).

**CONCLUSION:**

Demographics and socioeconomic factors, particularly lack of insurance and non–English-speaking status, are correlated with poor clinical follow-up after endovascular treatment of cerebral aneurysms. Patients at high risk of loss to follow-up should be counseled before treatment about risk of aneurysm recurrence.

Cerebral aneurysms are the main etiology of spontaneous subarachnoid hemorrhage (SAH), which afflicts 9 patients per 100 000 per year with ∼35% mortality and significant disability in survivors.^1,2^ In patients who survive the rupture, aneurysm securement to prevent rebleeding is the key neurosurgical treatment, which is accomplished through either endovascular embolization or surgical clipping.^3^ In unruptured aneurysms, if high-risk features are present that impart an elevated risk of rupture, then similar treatments are indicated.^4^

In contemporary practice, cerebral aneurysms are commonly treated through endovascular approaches, given multiple trials demonstrating clinical efficacy.^4,5^ However, endovascular approaches, particularly coiling, have demonstrated a significantly higher aneurysm recurrence rate than surgical clipping leading to frequent need for re-treatment.^4,6^ Given the higher aneurysm recurrence rate after endovascular coiling, frequent follow-up with angiography or other imaging modalities to monitor for aneurysm recurrence or need for additional treatment is increasingly important for these patients, but access to care because of socioeconomic factors may limit necessary follow-up.^7^ Herein, we perform a retrospective single-center study of 937 patients after endovascular cerebral aneurysm embolization to determine what demographic and socioeconomic factors are associated with poor access to follow-up to identify which patients may be at uniquely high risk of untreated aneurysm recurrence after endovascular intervention.

## METHODS

### Study Design, Setting, and Patient Selection

In this retrospective observational study, patients were included with a diagnosis of cerebral aneurysm requiring endovascular intervention at University of Florida (UF) Health between 2006 and 2017. This study was performed at a single center with interventions performed by 4 different endovascular neurosurgeons. Inclusion criteria included age >18 years, unruptured or ruptured cerebral aneurysm treated by endovascular intervention, and survival to discharge. Endovascular interventions included aneurysm treatment via coil, stent, or stent + coil. Patients were excluded if they were discharged to hospice, if aneurysm was mycotic, or if there was confirmation in the chart of death within 30 days of discharge. All patients received guideline-recommended postaneurysm treatment care.^1,8^ All patients received standard appointment reminders for follow-up either electronically, by phone, or by mail with reminders.

The study was approved by the local institutional review board (IRB201900190), including a waiver of informed consent.

### Data Collection and Management

Data were extracted from electronic medical records for 5 years after endovascular aneurysm treatment and included demographic variables of age, sex, employment status, proficiency in English language, race, marital status, and type of health insurance. Additional demographic factors included for each patient were distance of home address from UF Health and estimated household income based on zip code (2021 Internal Revenue Service income database^9^). Aneurysm characteristics recorded for each patient included rupture status at time of treatment, endovascular intervention type (coil, stent, or coil + stent), and whether the aneurysm demonstrated recurrence (defined as an increase in Modified Raymond–Roy Classification) on follow-up imaging. For each patient, attendance at neurosurgery follow-up was recorded over 5 years after endovascular treatment including office visits or angiograms. All data were stored in the institutional REDCap^TM^ database in a confidential and coded fashion.^10^

### Statistical Analysis

Unpaired Student *t*-test, 1-way analysis of variance with Tukey's multiple comparisons test, log-rank test, or χ^2^ tests were used for univariate analysis and survival curve analysis for each variable compared with follow-up visits and years of follow-up.

All statistical analyses were conducted using Prism 9 (GraphPad Software). The SD is used as the measure of variation throughout the text, and additionally the 95% CI is provided when a *P* value is provided, while graphical data are presented as the mean ± SD unless otherwise stated. For representation of *P* values on graphs, typical convention is used where “*” indicates a *P* < .05, “**” indicates a *P* < .01, “***” indicates a *P* < .001, and “****” indicates a *P* < .0001; “ns” indicates the *P* value is nonsignificant as *P* > .05.

## Data availability

Anonymized data will be shared with any qualified investigator upon request.

## RESULTS

### Cohort Characteristics

**FIGURE 1.**
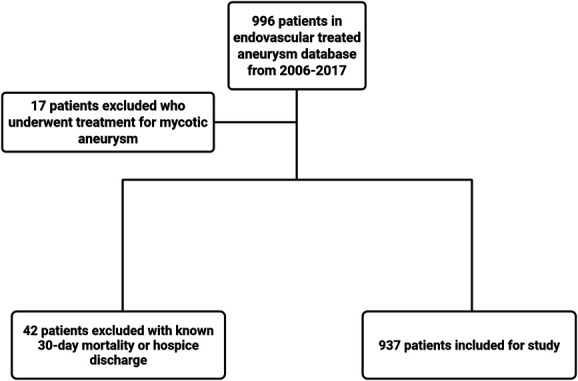
Diagram of patient inclusion and exclusion criteria with final cohort of included patients from a single center endovascular treatment database.

There were 937 patients who underwent endovascular embolization of a cerebral aneurysm from 2006 to 2017 meeting inclusion criteria (Figure 1). Institutional practice has a typical follow-up period of at least 5 years after treatment to ensure adequate treatment without recurrence; follow-up visits are either diagnostic angiograms or office visits with computed tomography angiogram or magnetic resonance angiogram. Cohort demographics and follow-up data are summarized in Table 1. The mean patient age was 56.6 ± 15.3 years, mean estimated household yearly income was $78 400 ± 118 000, and mean distance of home from treating hospital was 143 ± 227 miles. In the cohort, 265 patients (28%) never returned for a follow-up appointment, 469 patients (51%) had partial follow-up, and 203 patients had complete follow-up of 5+ years (22%). Of treated patients, 415 (45%) presented initially with aneurysm rupture while 522 (55%) were unruptured at the time of treatment. For endovascular intervention, 574 (61%) underwent coil embolization, 103 (11%) were treated with stent embolization, 260 (28%) underwent combination stent + coiling. Of patients in this database, 141 (14%) were known to have aneurysm recurrence as evidenced by an increase in Modified Raymond–Roy Classification on subsequent vascular imaging, while the remainder had no recurrence or status was unknown.

**TABLE 1.** Demographic Variables and Follow-up at 5 y

Variable	Total cohort	No follow-up	Partial follow-up	Completed follow-up	T or ANOVA test (*P* value)
Patients included (N)	937	265 (28%)^a^	469 (50%)	203 (22%)	—
Age (y)	56.6 ± 15.3^b^	56.0 ± 18.0	56.9 ± 15.0	56.5 ± 12.0	.99 ns
Estimated household yearly income ($)	$78 400 ± 118 000	$81 300 ± 84 900	$79 900 ± 139 000	$70 900 ± 54 900	.99 ns
Distance of home from treating hospital (miles)	143 ± 227	214 ± 338	123 ± 168	98 ± 88	<.0001****

ANOVA, analysis of variance.

a Number of individuals (N) and their percentage of the total sample (n/N). For representation of *P* values, typical convention is used where * indicates a *P* < .05, ** indicates a *P* < .01, *** indicates a *P* < .001, and **** indicates a *P* < .0001; ns indicates the *P* value is nonsignificant as *P* > .05.

b Mean value and SD.

### Continuous Variables and Relationship With Follow-up

**FIGURE 2.**
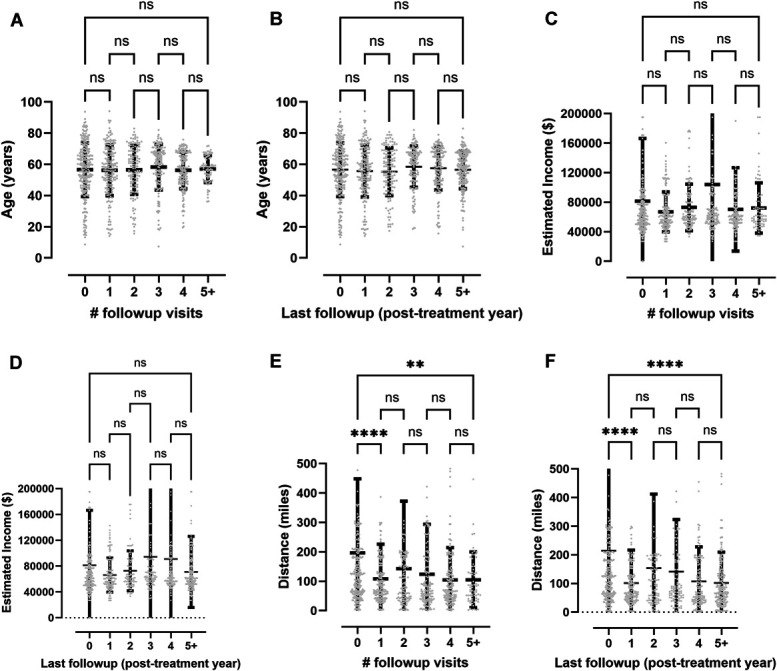
Relation between select demographics and attendance at clinical follow-up. **A**, Plot of age for patients and number of follow-up visits attended. **B**, Plot of age for patients and number of years until lost to follow-up. **C**, Plot of estimated household income (based on zip code) for patients and number of follow-up visits attended. **D**, Plot of estimated household income (based on zip code) for patients and number of years until lost to follow-up. **E**, Plot of distance lived in miles from treating hospital for patients and number of follow-up visits attended. **F**, Plot of distance lived in miles from treating hospital for patients and number of years until lost to follow-up. All error bars are SD. One-way analysis of variance. test with Tukey's multiple comparisons test shown for all statistical tests with *P*-values indicated. For representation of *P* values on graphs, typical convention is used where * indicates a *P* < .05, ** indicates a *P* < .01, *** indicates a *P* < .001, and **** indicates a *P* < .0001; ns indicates the *P* value is nonsignificant as *P* > .05.

Variables including age, estimated yearly household income, and distance of home from treating hospital were analyzed with univariate one-way analysis of variance with Tukey's test to determine association of these variables with both number of follow-up visits and length of time until lost to follow-up (Figure 2). For age (Table 1, Figure 2A and 2B), there was no significant difference in total number of follow-up visits (56.0 ± 18.0 years [95% CI ± 2.2] for no follow-up vs 57.1 ± 8.6 years [95% CI ± 1.2] for 5+ follow-up visits, *P* > .99) or time until lost to follow-up (56.0 ± 18.0 years [95% CI ± 2.2] for no follow-up vs 56.6 ± 12 years [95% CI ± 1.7] for 5+ years' follow-up, *P* > .99). There was also no difference in estimated yearly household income between these groups ($81 300 ± 84 900 [95% CI ± 10 200] for no follow-up vs $70 900 ± 54 900 for 5+ years' follow-up [95% CI ± 7500], *P* > .99; Table 1, Figure 2C and 2D). Patients with no follow-up lived a significantly greater distance from the treating hospital than those in the groups with 1 or more follow-up within any time frame, and similarly for patients with 1 or more years of follow-up (214 ± 338 miles [95% CI ± 40] for no follow-up vs 98 ± 88 miles [95% CI ± 12] for 5+ years follow-up, *P* < .0001; Table 1, Figure 2E and 2F).

### Demographic Variables and Relationship With Follow-up

**FIGURE 3.**
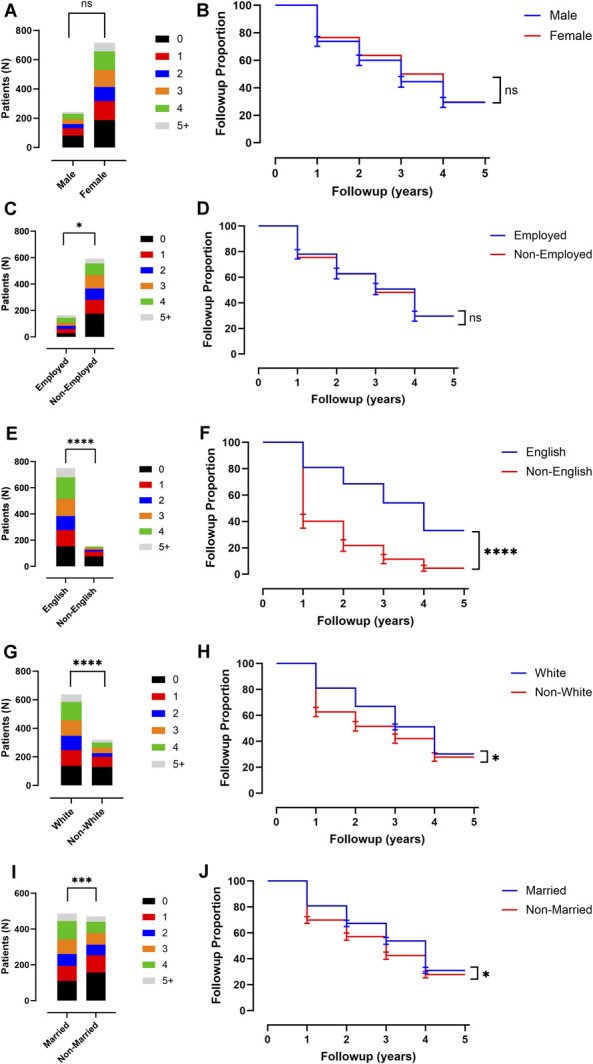
Correlation between select demographics and socioeconomic variables with attendance at clinical follow-up. **A**, Plot of sex of patients and proportion of follow-up visits attended. **B**, Kaplan–Meier curve of sex of patients and number of years until lost to follow-up. **C**, Plot of employment status of patients and proportion of follow-up visits attended. **D**, Kaplan–Meier curve of employment status of patients and number of years until lost to follow-up. **E**, Plot of primary spoken language of patients and proportion of follow-up visits attended. **F**, Kaplan–Meier curve of primary spoken language of patients and number of years until lost to follow-up. **G**, Plot of race of patients and proportion of follow-up visits attended. **H**, Kaplan–Meier curve of race of patients and number of years until lost to follow-up. **I**, Plot of marital status of patients and proportion of follow-up visits attended. **J**, Kaplan–Meier curve of marital status of patients and number of years until lost to follow-up. All error bars are SD. χ^2^ test is shown for all proportion of follow-up visit plots and log-rank test is shown for all Kaplan–Meier curves. *P*-values are indicated. For representation of *P* values on graphs, typical convention is used where * indicates a *P* < .05, ** indicates a *P* < .01, *** indicates a *P* < .001, and **** indicates a *P* < .0001; ns indicates the *P* value is nonsignificant as *P* > .05.

Demographic variables including sex, employment status, spoken language, race, and marital status were analyzed with univariate χ^2^ tests or survival curve comparison with log-rank test to determine association of these variables with number of follow-up visits and length of time until lost to follow-up, respectively (Table 2, Figure 3). For sex, there was no significant difference in mean number of follow-up visits (1.7 ± 1.6 visits [95% CI ± 0.2] for men and 2.1 ± 1.7 visits [95% CI ± 0.1] for women, *P* = .12; Table 2, Figure 3A) or in time until lost to follow-up (2.0 ± 1.9 years [95% CI ± 0.2] for men and 2.4 ± 1.9 years [95% CI ± 0.1] for women, *P* = .17; Table 2, Figure 3B). For employment status, there was significantly less follow-up for nonemployed patients in mean number of follow-up visits (2.3 ± 1.7 visits [95% CI ± 0.3] for employed and 1.9 ± 1.8 visits [95% CI ± 0.1] for nonemployed, *P* = .012; Table 2, Figure 3C), but not in time until lost to follow-up (2.6 ± 1.9 years [95% CI ± 0.3] for employed and 2.2 ± 2.0 years [95% CI ± 0.2] for nonemployed, *P* = .83; Table 2, Figure 3D). For spoken language, there was significantly less follow-up for non–English-speaking patients in both mean number of follow-up visits (2.3 ± 1.7 visits for English-speaking [95% CI ± 0.1] and 0.74 ± 1.0 visits [95% CI ± 0.2] for non–English-speaking, *P* < .0001; Table 2, Figure 3E) and in time until lost to follow-up (2.6 ± 1.9 years [95% CI ± 0.1] for English-speaking and 0.82 ± 1.2 years [95% CI ± 0.2] for non–English-speaking, *P* < .0001; Table 2, Figure 3F). For race, there was significantly less follow-up for non-White patients in both mean number of follow-up visits (2.2 ± 1.6 visits [95% CI ± 0.1] for White and 1.5 ± 1.6 visits [95% CI ± 0.2] for non-White, *P* < .0001; Table 2, Figure 3G) and in time until lost to follow-up (2.6 ± 1.9 years [95% CI ± 0.2] for White and 1.7 ± 1.9 years [95% CI ± 0.2] for non-White, *P* = .034; Table 2, Figure 3H). For marital status, there was significantly less follow-up for nonmarried patients in both mean number of follow-up visits (2.2 ± 1.7 visits [95% CI ± 0.2] for married and 1.7 ± 1.6 visits [95% CI ± 0.2] for nonmarried, *P* = .0003; Table 2, Figure 3I) and in time until lost to follow-up (2.6 ± 1.9 years [95% CI ± 0.2] for married and 2.0 ± 1.9 years [95% CI ± 0.2] for nonmarried, *P* = .030; Table 2, Figure 3J).

**TABLE 2.** Demographic Variables, Number of Follow-up Visits, and Time Until Lost to Follow-up

Variable	Total cohort	Average follow-up visits (N)	χ^2^ test (*P* value)	Average time until lost to follow-up (y)	Log-rank test (*P* value)
Patients included (N)	937	2.0 ± 1.7^a^	—	2.3 ± 1.8	—
Male	227	1.7 ± 1.6	.12 ns	2.0 ± 1.9	.17 ns
Female	710	2.1 ± 1.7		2.4 ± 1.9	
Employed	162^b^	2.3 ± 1.7	.012*	2.6 ± 1.9	.83 ns
Nonemployed/retired	590	1.9 ± 1.8		2.2 ± 2.0	
English-speaking	749^b^	2.3 ± 1.7	<.0001****	2.6 ± 1.9	<.0001****
Non–English-speaking	140	0.74 ± 1.0		0.82 ± 1.2	
White race	619^b^	2.2 ± 1.6	<.0001****	2.6 ± 1.9	.034*
Non-White race	270	1.5 ± 1.6		1.7 ± 1.9	
Married	479^b^	2.2 ± 1.7	.0003***	2.6 ± 1.9	.030*
Not married	419	1.7 ± 1.6		2.0 ± 1.9	
Aneurysm ruptured	415	1.5 ± 1.6	<.0001****	1.7 ± 1.9	.037*
Aneurysm nonruptured	522	2.4 ± 1.6		2.7 ± 1.9	
Insured	641^b^	2.5 ± 1.7	<.0001****	2.9 ± 1.9	<.0001****
Noninsured	139	1.0 ± 1.2		1.1 ± 1.4	
Private insurance	161	2.4 ± 1.7	.32 ns	2.8 ± 2.0	.60 ns
Government insurance	480	2.5 ± 1.6		2.9 ± 1.9	

a Mean value and SD.

b Data were not available for all individuals for these variables. For representation of *P* values on graphs, typical convention is used where * indicates a *P* < .05, ** indicates a *P* < .01, *** indicates a *P* < .001, and **** indicates a *P* < .0001; ns indicates the *P* value is nonsignificant as *P* > .05.

### Insurance Status, Type, and Relationship With Follow-up

**FIGURE 4.**
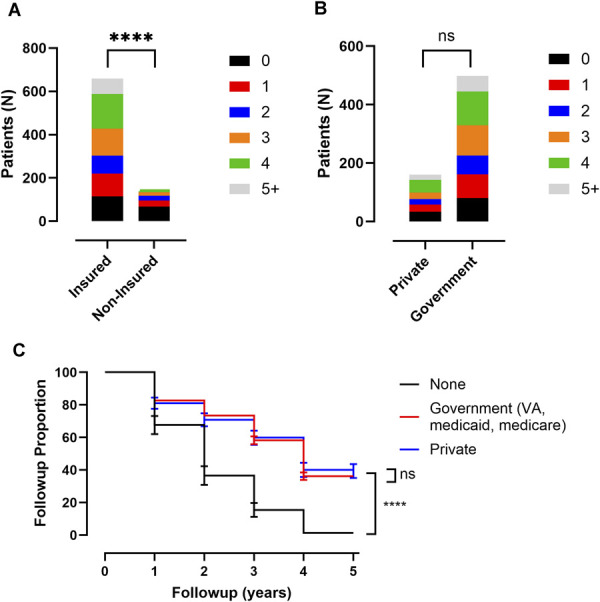
Correlation between insurance status with attendance at clinical follow-up. **A**, Plot of insurance status of patients and proportion of follow-up visits attended. **B**, Plot of insurance type of patients and proportion of follow-up visits attended. **C**, Kaplan–Meier curve of insurance status and type for patients and number of years until lost to follow-up. All error bars are SD. χ^2^ test is shown for all proportion of follow-up visit plots and log-rank test is shown for all Kaplan–Meier curves. *P*-values are indicated. For representation of *P* values on graphs, typical convention is used where * indicates a *P* < .05, ** indicates a *P* < .01, *** indicates a *P* < .001, and **** indicates a *P* < .0001; ns indicates the *P* value is nonsignificant as *P* > .05.

Insurance status along with type of insurance were analyzed with univariate χ^2^ tests, or survival curve comparison with log-rank test to determine association of these variables with number of follow-up visits and length of time until lost to follow-up, respectively (Table 2, Figure 4). Concerning insurance status, there was significantly less follow-up for noninsured patients in both mean number of follow-up visits (2.5 ± 1.7 visits [95% CI ± 0.1] for insured patients and 1.0 ± 1.2 visits [95% CI ± 0.2] for noninsured, *P* < .0001; Table 2, Figure 4A) and in time until lost to follow-up (2.9 ± 1.9 years [95% CI ± 0.1] for insured and 1.1 ± 1.4 years [95% CI ± 0.2] for noninsured, *P* < .0001; Table 2, Figure 4C). For type of insurance, government payer (Medicare, Medicaid, Veteran's association) compared with private payer, there was no significant difference in mean number of follow-up visits (2.4 ± 1.7 visits [95% CI ± 0.3] for private and 2.5 ± 1.6 visits [95% CI ± 0.1] for government, *P* = .32; Table 2, Figure 4B) or in time until lost to follow-up (2.8 ± 2.0 years [95% CI ± 0.3] for private and 2.9 ± 1.9 years [95% CI ± 0.2] for government, *P* = .60; Table 2, Figure 4C).

### Aneurysm Rupture Status at Presentation and Relationship With Follow-up

**FIGURE 5.**
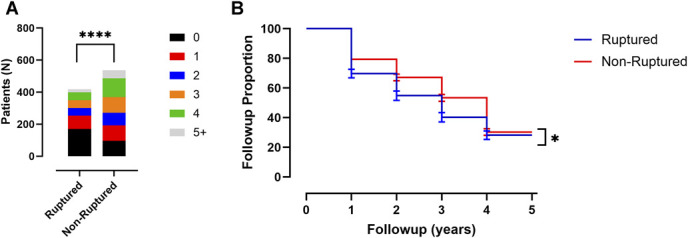
Correlation between aneurysm rupture status with attendance at clinical follow-up. **A**, Plot of aneurysm rupture status of patients and proportion of follow-up visits attended. **B**, Kaplan–Meier curve of aneurysm rupture status of patients and number of years until lost to follow-up. All error bars are SD. χ^2^ test is shown for all proportion of follow-up visit plots and log-rank test is shown for all Kaplan–Meier curves. *P*-values are indicated. For representation of *P* values on graphs, typical convention is used where * indicates a *P* < .05, ** indicates a *P* < .01, *** indicates a *P* < .001, and **** indicates a *P* < .0001; ns indicates the *P* value is nonsignificant as *P* > .05.

Aneurysm rupture status at the time of endovascular treatment was analyzed with univariate χ^2^ tests, or survival curve comparison with log-rank test to determine association of this variable with number of follow-up visits and length of time until lost to follow-up, respectively (Table 2, Figure 5). For rupture status, there was significantly less follow-up for patients with aneurysm rupture at presentation in both mean number of follow-up visits (1.5 ± 1.6 visits [95% CI ± 0.2] for ruptured patients and 2.4 ± 1.6 visits [95% CI ± 0.1] for nonruptured, *P* < .0001; Table 2, Figure 5A) and in time until lost to follow-up (1.7 ± 1.9 years [95% CI ± 0.2] for ruptured and 2.7 ± 1.9 years [95% CI ± 0.2] for nonruptured, *P* = .037; Table 2, Figure 5B).

## DISCUSSION

### Key Results

The decision-making process for treating intracranial aneurysms is multifaceted, with most surgeons focusing on aneurysm characteristics including size, location, shape, etc. in determining whether observation, endovascular embolization, or surgical clipping is most appropriate.^4,11^ However, patient outcomes may be influenced by their ability to adhere to follow-up care, and the decision to observe high-risk aneurysms, or perform endovascular coiling in which recurrence rates are as high as ∼18%,^5,11^ may lead to detrimental outcomes in patients with high risk for loss to follow-up. Treatment decisions need to be made in the context of each patient's barriers to care as well as aneurysm characteristics, and proper support systems must be in place before treatment if possible. Social and economic barriers are significantly associated with likelihood of consistent follow-up as discussed in this study. Particularly, lack of insurance and non–English-speaking status are strongly correlated with poor follow-up after endovascular treatment, suggesting barriers to navigating the health care system as driving features. Although race, marital status, and employment status were associated with decreased follow-up, the effect size was far less than language or insurance status. Age and sex were not associated with likelihood of clinical follow-up. In combination, these findings may suggest that discriminatory factors based on demographics are not as important in determining likelihood of clinical follow-up so much as access to care itself, which is likely limited in patients who lack insurance or do not speak English. These findings are underscored by aneurysm rupture at presentation having less of an effect on follow-up than insurance or language, as aneurysmal rupture is expected to result in disability in survivors, which expectedly limits access to follow-up. Although estimated household income was not associated with likelihood of follow-up, the study was limited to using zip code as the surrogate for this measure.

### Interpretation

Current literature describes race and insurance status as factors relating to increased mortality and poorer functional outcomes after aneurysmal SAH.^12^ However, studies have yet to probe into the reason for these disparities. In this study, we show that insurance status, speaking English, and distance from treatment center are all factors that strongly affect the probability of follow-up after endovascular treatment of an intracranial aneurysm. We hypothesize that the differences in the number of follow-up visits and length of follow-up after endovascular aneurysm treatment in different socioeconomic groups are due to the ability to access medical care based on the pattern of significant factors discussed. These results add to literature highlighting the health-related social needs of patients with cerebral aneurysms and the disparities that must be considered in the planning of aneurysm treatment.^7^

Insurance status is a key driver of outcomes in the treatment of cerebral aneurysms and SAH. A previous study has shown patients with private insurance are 5 times more likely to have an aneurysm treated than patients without insurance and 3 times as likely to be treated for aneurysmal SAH.^7^ Our results on insurance status affecting follow-up reflect the financial commitment that patients must make to continue surveillance angiograms after coiling. We found no significant difference in aneurysm surveillance follow-up between private- and government-insured groups, supporting the hypothesis that government insurance programs are noninferior in the realm of postoperative care for intracranial aneurysms.

Despite translation services being available at our institution, our study found a decrease in follow-up rates in non-English speakers. This highlights the barrier that arises when a surgeon or care team and the patient do not speak the same language.^13^ Surgeons must be watchful for other disparities, such as insurance status, which are more common in patients with limited English proficiency and include these factors into their plan of care.^14^

Distance from a treatment center is an important factor that we determined affects the probability of continued follow-up after intracranial aneurysm coiling. Other research has found that patients with aneurysmal SAH living more than 20 miles from a treatment center have a 12% greater absolute mortality rate than those living less than 20 miles from a treatment center.^15^ The bulk of this increased mortality is surely because of delay to treatment, but other factors, such as the decreased probability of continued follow-up shown in our study, may also affect long-term SAH mortality.

**FIGURE 6.**
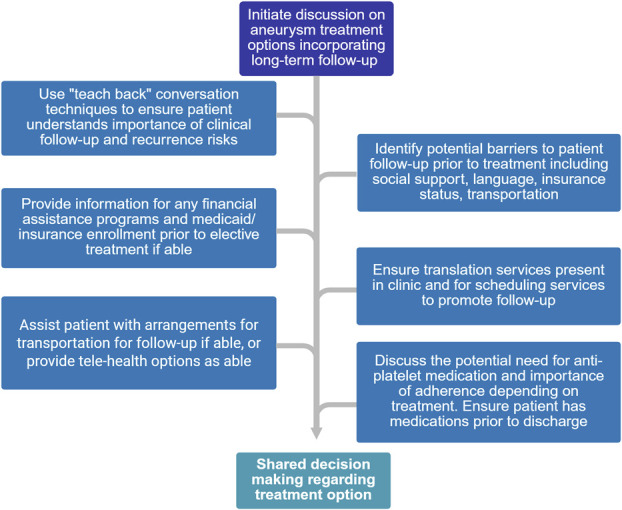
Flow diagram with key discussion points to identify barriers to follow-up in shared decision making with patient concerning therapeutic options for aneurysms for which treatment is recommended.

Our results reflect the complex health-related social and economic needs of patients undergoing aneurysm treatment. Treatment of cerebral aneurysms and the subsequent hospital stay is expensive, with one study finding between years 2013-2015 cost estimates of $85 553 for coiling and $74 192 for clipping.^16^ The need for continuous endovascular procedures and potential expensive antiplatelet medications after endovascular stenting adds additional barriers for patients with socioeconomic disadvantages that may prevent successful follow-up. Indeed, barriers to attainment of antiplatelet medication indicated for clopidogrel nonresponders such as ticagrelor can lead to fatal stent occlusion, and it is critical that patient access to proper post-treatment medications be confirmed before treatment. Based on the results of our investigation, we recommend surgeons consider an individual's barriers to care when treating patients. A patient's care team must prioritize discussions about these socioeconomic barriers with the patient, explain the consequences of nonadherence to clinical follow-up, and ensure methods to assist are in place before treatment to improve outcomes for endovascular treatment of cerebral aneurysms^17^ (Figure 6).

### Limitations

This is a single-center retrospective observational study for which generalization of results to patients at other institutions and practices is inherently limited. Loss to follow-up analysis could be limited by mortality or patients transferring care to other facilities that is not captured in the medical record. Patients may also have not received the same type of reminders for follow-up appointments, which could bias results. Language and insurance status having a larger clinical effect size than aneurysm rupture status suggests that mortality is not a leading driver of loss to follow-up.

## CONCLUSION

Demographics and socioeconomic variables, particularly lack of insurance and not speaking English, are correlated with decreased attendance at clinical follow-up visits after endovascular treatment of cerebral aneurysms. Patients at high risk of loss to follow-up should be intensively counseled before endovascular treatment on importance of follow-up, and identifiable barriers should be addressed.

## COMMENTS

The authors are commended for their rigorous study of the demographics and socioeconomic factors that condition quality of the clinical follow-up after endovascular treatment of cerebral aneurysms. Lack of insurance and not speaking English were found determinant in loss of proper follow-up. As stressed by the authors, these pejorative factors have to be taken into account before deciding treatment. These conditions, likely linked to the patient's precarious social conditions and/or poor educative background, should incite to provide additional assistance to these vulnerable patients.

For prediction of recurrence, and consequently risk of potential bleeding/rebleeding, the authors have adopted the Modified Raymond–Roy Classification (MRRC) adapted from the Raymond–Roy Occlusion Classification (RROC).

To be recalled, the RROC, which was for long the standard for evaluating coiled aneurysms, described Class I as complete obliteration, Class II as residual neck, and Class III as residual aneurysm.^1a^

The MRRC was conceived to further differentiate Class III aneurysms into those likely to progress to complete occlusion and those likely to remain incompletely occluded or to worsen.^2a^ In this classification, Class I is complete obliteration, Class II is residual neck, Class IIIa is residual aneurysm with contrast within coil interstices, and Class IIIb is residual aneurysm with contrast along aneurysm wall, this later class with more propensity to bleed/rebleed.

In the presented observational study of 937 patients, the percentage of aneurysms considered having recurred was calculated at 14%. This % is well in the range of the publications on endovascular techniques, and also of most of the reports on the clipping techniques, 5.9% in our own series of 305 clipped cases.^3a^ In our article, aneurysmal remnants were classified into 5 grades. Grades I and II corresponded to only a neck remnant, with less (Grade I) or more (grade II) than 50% of neck size, both of those grades only justifying repeated angiographic controls. Grade III corresponded to a residual lobe of a multilobulated sac, Grade IV to a residual sac of less, and Grade V of more than 75% of the aneurysmal size. In all those, reoperation or complementary endovascular obliteration was considered.

**Marc Sindou**

*Lyon, France*
